# Two-Channel Graphene pH Sensor Using Semi-Ionic Fluorinated Graphene Reference Electrode

**DOI:** 10.3390/s20154184

**Published:** 2020-07-28

**Authors:** Dae Hoon Kim, Woo Hwan Park, Hong Gi Oh, Dong Cheol Jeon, Joon Mook Lim, Kwang Soup Song

**Affiliations:** 1 Department of Medical IT Convergence Engineering, Kumoh National Institute of Technology, Gumi 39177, Korea; dhoonkim@kumoh.ac.kr (D.H.K.); parkwh123@gmail.com (W.H.P.); oh558@naver.com (H.G.O.); vcaptin@kumoh.ac.kr (D.C.J.); 2 Department of Creative Convergence Engineering, Hanbat National University, Daejeon 34158, Korea; jmlim@hanbat.ac.kr

**Keywords:** graphene, fluorinated graphene, pH sensor, reference electrode

## Abstract

A reference electrode is necessary for the working of ion-sensitive field-effect transistor (ISFET)-type sensors in electrolyte solutions. The Ag/AgCl electrode is normally used as a reference electrode. However, the Ag/AgCl reference electrode limits the advantages of the ISFET sensor. In this work, we fabricated a two-channel graphene solution gate field-effect transistor (G-SGFET) to detect pH without an Ag/AgCl reference electrode in the electrolyte solution. One channel is the sensing channel for detecting the pH and the other channel is the reference channel that serves as the reference electrode. The sensing channel was oxygenated, and the reference channel was fluorinated partially. Both the channels were directly exposed to the electrolyte solution without sensing membranes or passivation layers. The transfer characteristics of the two-channel G-SGFET showed ambipolar field-effect transistor (FET) behavior (p-channel and n-channel), which is a typical characteristic curve for the graphene ISFET, and the value of V*_Dirac_* was shifted by 18.2 mV/pH in the positive direction over the range of pH values from 4 to 10. The leakage current of the reference channel was 16.48 nA. We detected the real-time pH value for the two-channel G-SGFET, which operated stably for 60 min in the buffer solution.

## 1. Introduction

Since the invention of the ion-sensitive field-effect transistor (ISFET) by Bergveld (1970), field-effect transistor (FET)-based ion sensors have been widely studied for detecting many types of specific ions [1]. The ion-sensitive field-effect transistor (ISFET) has advantages such as high sensitivity, rapid response, high input impedance, low output impedance, miniaturization, and low cost. In the ISFET sensing system, a durable reference electrode is required to set a precise and stable potential reference to the ISFET measured solution [2,3,4,5]. Conventionally, the Ag/AgCl electrode has been used as the reference electrode in the ISFET sensing system [6,7,8,9]. However, the Ag/AgCl reference electrode limits the advantages of the ISFET, especially miniaturization and low cost. The Ag/AgCl reference electrode is difficult to miniaturize to micrometer size, and its fabrication is incompatible with the semiconductor technology used to manufacture ISFETs [10]. Some studies have reported methods using solid-state reference electrodes or back gates to replace the Ag/AgCl reference electrode [10,11]. However, these methods are incompatible with semiconductor fabrication technology and have reliability issues. Therefore, finding a robustly integrated reference electrode remains the most crucial factor for FET-based pH sensors.

Graphene, a single-atom layer with a two-dimensional (2D) hexagonal structure of carbon, has high electrical conductivity, physical stability, and unique chemical characteristics [12,13,14]. Recently, large-area graphene sheets grown by the chemical vapor deposition (CVD) method were transferred onto silicon wafer, glass, and polyethylene terephthalate (PET) [15]. Such graphene sheets are commercialized and used in bioengineering to fabricate biosensors. Graphene-based FET biosensors, which are highly sensitive and selective, were developed with the aim of detecting biomolecules [16,17,18].

In this work, we fabricated a two-channel graphene solution gate field-effect transistor (G-SGFET) to detect the pH of an electrolyte solution. We integrated two types of functionalized graphene channels into one device to fabricate the two-channel G-SGFET. One graphene channel was the sensing channel similar to ISFET (G-ISFET), and the other graphene channel was the reference channel substituting the Ag/AgCl reference electrode. The structure of the two-channel G-SGFET was a two-dimensional (2D) structure. The reference channel is directly exposed to the electrolyte solution without passivating membranes, which are necessary for the silicon-based reference field-effect transistor (REFET), to set up a precise and stable potential with respect to the sensing channel in the electrolyte solution. The sensing channel of the two-channel G-SGFET detects the concentration of proton ([H^+^]). The pH sensitivity, real-time detection, and long-term stability of the two-channel G-SGFET are evaluated by current–voltage (I–V) measurements in the electrolyte solution. The two-channel G-SGFET overcomes the limitations of the G-ISFET.

## 2. Materials and Methods

### 2.1. Fabrication of G-SGFET

Large-sized graphene sheets on PET substrate were purchased from MCK Tech Co. (Ansan, Korea). The application of graphene sheets onto PET substrates has potential for use in bioscience owing to the flexibility of the substrate. Gold (Au) was evaporated in a vacuum chamber (5.0 × 10^−6^ Torr) using a thermal evaporator to form the drain and source electrodes on the graphene sheet. The thickness of the gold electrode was 200 nm, and the gate channel was 5 mm in width and 500 μm in length. To apply bias to the electrodes, conductive wires were bonded using silver paste on the drain and source electrodes. Finally, the drain and source electrodes were covered with epoxy resin (HE 205 from Malaysia) to protect the electrodes from the electrolyte. The transfer characteristics of the two-channel G-SGFET were measured using two digital source meters (KEITHLEY 2400, Cleveland, OH, USA). All electrical measurements were carried out at 28 °C and biased within the potential window of graphene to prevent the redox reaction on the graphene gate channel. The drain–source voltage (*V_DS_*) was fixed at 0.05 V and the gate–source voltage (*V_GS_*) was swept from 0.0 to 1.0 V. The sensitivity of the pH was evaluated by the shift in the voltage of the Dirac point (*ΔV_Dirac_*) in the G-ISFET. Carmody buffer solution (0.2 M boric acid, 0.05 M citric acid, and 0.1 M tri-sodium phosphate) was used as the pH buffer solution, with the pH adjusted from 4 to 10. Ultrapure water (18.2 MΩ·cm) was used for the preparation of all the solutions.

### 2.2. Functionalization of Graphene

The graphene sheet was functionalized by plasma treatment, employing a 50 kHz radio frequency plasma source at 50 W using a plasma generator (CUTE, Femto Science Co. Ltd., Hwaseong-Si, Korea). The plasma treatment was carried out at 28 °C (gas pressure fixed at 1 × 10^−2^ Torr) on the graphene gate channel in an O_2_ gas environment to oxidize for 10 s or in a C_3_F_8_ gas environment to fluorinate for 20 min. The O_2_ and C_3_F_8_ gas flow rates were maintained at 20 and 10 sccm, respectively. Thereafter, the fluorinated graphene gate channel was exposed to air at 28 °C and left to stabilize for 72 h.

A spatially resolved X-ray photoelectron spectroscope (XPS; Thermo Fisher, Waltham, MA, USA) was used for determining the surface composition and bonding state of the functionalized graphene sheets with a monochromatic Al Kα (1486.6 eV) X-ray source. The beam diameter was set to 400 μm, and survey scans using a resolution of 10 scans were performed on the graphene that was transferred onto the SiO_2_/Si substrate. The properties of the functionalized graphene were characterized by Raman spectroscopy (Renishaw, system1000, Gloucestershire, UK) using an argon-ion laser at an excitation wavelength of 514 nm and a spot size of 1 μm. All Raman measurements were performed at room temperature.

## 3. Results and Discussion

### 3.1. Surface Analysis of Graphene

**Figure 1:**
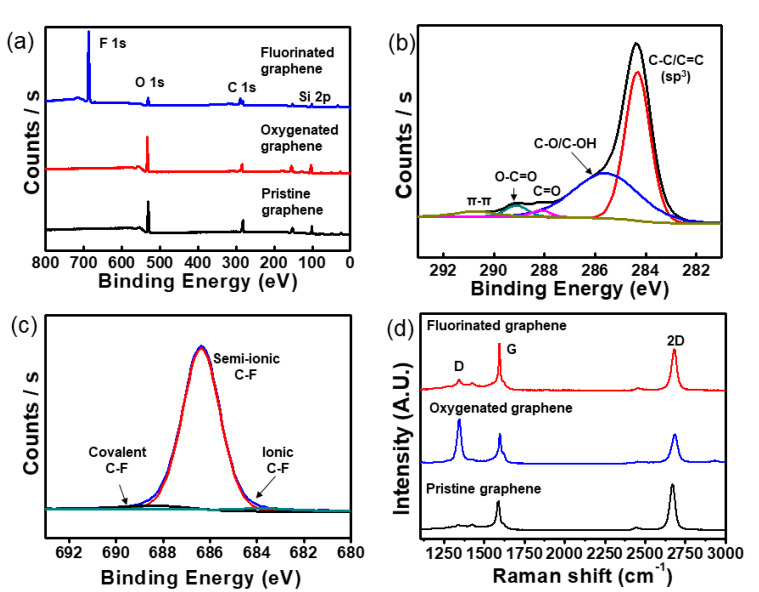
XPS and Raman spectra of graphene. (**a**) Survey spectra of XPS on pristine, oxygenated and fluorinated graphene; (**b**) deconvoluted C 1s peaks on partially oxygenated graphene; (**c**) deconvoluted F 1s peaks on partially fluorinated graphene; (**d**) Raman spectra of pristine, oxygenated and fluorinated graphene.

The survey scans of XPS spectrum on the pristine, oxygenated and fluorinated graphene sheets are shown in Figure 1a. The atomic ratio of oxygen atoms was increased to 24.07% on the oxygenated graphene sheets, as compared to that of pristine graphene (21.42%), using plasma treatment for 10 s. The main carbon C 1s from the oxygenated graphene was deconvoluted with four components that denoted carbon atoms in four specific functional groups [19]. The four peaks were associated with the graphitic peak (C-C/C=C), hydroxyl groups (C-OH), carbonyl groups (C=O), carboxyl groups (O=C-OH) at 284.67, 286.6, 288.9 and 290.67 eV, respectively. A satellite of the graphitic peak (π-π) was also found at 291.72 eV, as shown in Figure 1b. The main carbon C 1s from the pristine and fluorinated graphene were shown in our previous work [20].

Fluorinated graphene has received attention in a variety of applications, including self-cleaning, super-hydrophobic coatings and electrochemical electrodes, because fluorinated graphene has low surface energy, high chemical stability and temperature stability [19,21,22]. The plasma treatment in fluorine gas (SF_6_, CF_4_, C_4_F_8_, and C_3_F_8_) environment is considered a clean method for manufacturing fluorinated graphene [19,22]. The fluorinated graphene has three types of bonding properties, such as ionic, semi-ionic, and covalent C-F bonds depending on the plasma condition [23]. These bonding properties are characterized with XPS spectrum analysis. The deconvolution of F 1s peak defines the three types of C-F bonds; ionic (684 eV), semi-ionic (686–687 eV), and covalent bond (689–691 eV) [23,24]. When the graphene sheet was functionalized with fluorine for 20 min, the atomic ratio of fluorine was 49.8% and the content of oxygen clearly decreased because the oxygen atoms were substituted by carbon or fluorine, as shown in Figure 1b and Figure S1. The main fluorine F 1s from fluorinated graphene was deconvoluted with three components that denoted fluorine atoms in three specific functional groups [22]. The three peaks were associated with the ionic bond (2.8%) at 684.8 eV, semi-ionic bond (93.8%) at 687.3 eV, and covalent bond (3.4%) at 689.4 eV, respectively, as shown in Figure 1c. As a result of plasma treatment for 20 min in a C_3_F_8_ gas environment, the graphene sheet was functionalized with three types of fluorine bonds, and the dominant type of fluorine bond was semi-ionic C-F bonding. The ionic or semi-ionic C-F bonds on graphene sheet have conductivity like a metal or semiconductor, respectively [22], and the covalent C-F bond on the graphene sheet is an insulator [24].

The Raman spectra of the pristine, oxygenated and fluorinated graphene sheets are shown in Figure 1d. The G and 2D peaks of the pristine graphene sheet were centered at 1585 and 2700 cm^−1^, respectively. The D peak caused by defects at 1358 cm^−1^ was observed. The area intensity ratio (I_G_/I_2D_) of pristine was 0.4. The pristine graphene sheet was monolayer [25]. After fluorination using plasma treatment, the G and 2D peaks shifted 1589 and 2709 cm^−1^. The I_G_/I_2D_ was 0.46 and the intensity of the D peak was slightly greater than that of the pristine graphene. However, the I_G_/I_2D_ of oxygenated graphene was 0.55 and the D peak was highly increased. After oxygenation, D’ peak appears at ~1620 cm^−1^, which originate from double-resonance processes at the K point in the presence of defects intravalley phonon. The increased D peak of oxygenated graphene was partially etched and the defect was increased by the plasma oxygenation process. The full width at half-maximum (FWHM) of the 2D peak of pristine and fluorinated graphene was ~36 cm^−1^, and after oxygenation this increased to ~41 cm^−1^. The hall measurement was conducted to evaluate the conductivity of the pristine, fluorinated and oxygenated graphene sheets. The sheet resistance of the pristine and fluorinated graphene sheets was 475 and 603 Ω/sq, respectively. The sheet resistance was increased by fluorination due to the covalent C-F bond (3.4%), which was consistent with XPS and Raman spectra. The sheet resistance of the oxygenated graphene sheet was 1.99 KΩ/sq. The high sheet resistance of oxygenated graphene was due to the change in the carbon structure from an sp^2^-hybridized carbon structure to an sp^3^-hybridized carbon structure by the oxygenation.

### 3.2. The pH Sensitivity in Each Functionalized G-ISFET

**Figure 2:**
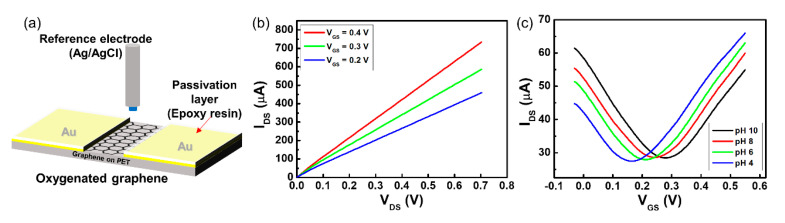
Typical three-dimensional pH sensor using graphene field-effect transistor. (**a**) Schematic illustration of G-ISFET with Ag/AgCl reference electrode. For oxygenated G-ISFET: (**b**) *I_DS_*–*V_DS_* transfer characteristic with respect to *V_GS_* and (**c**) *I_DS_*–*V_GS_* transfer characteristic with respect to pH value.

Although the two-channel G-SGFET was fabricated for pH sensing without a reference electrode, each functionalized G-ISFET works like a conventional ISFET with an Ag/AgCl reference electrode. As shown in Figure 2a, the pH sensitivity of each functionalized G-ISFET was evaluated. The G-ISFET is characterized by the drain–source current (*I_DS_*), drain–source voltage (*V_DS_*), and gate–source voltage (*V_GS_*) in the electrolyte solution. After the pH buffer exchange, the sensor was stabilized for 2 min before the steady-state electrical measurements of the transfer characteristics were conducted. To obtain the *I_DS_–V_DS_* characteristics of the oxygenated graphene channel, *V_DS_* was swept from 0.0 to 0.7 V in a buffer solution of pH 8. *I_DS_* increased with respect to *V_GS_* at the n-channel region, as shown in Figure 2b.

The strength of *I_DS_* in the oxygenated gate channel was low compared to the strength of *I_DS_* in the pristine gate channel (not shown). The conductivity of the oxidized gate channel was decreased by partial substitution from the sp^2^-hybridized carbon structure to the sp^3^-hybridized carbon structure by oxygenation of the graphene sheet, and an amorphous carbon structure exists due to the collision of oxygen ions on the oxidized gate channel surface after plasma treatment [26]. The sp^3^-hybridized carbon structure on the graphene sheet is an insulator [27,28]. *V_DS_* was fixed at 0.05 V and *V_GS_* was swept from 0.0 to 0.6 V to assess the *I_DS_–V_GS_* characteristics of the G-ISFET in the pH buffer solution. The value of *V_GS_* at the lowest value of *I_DS_* is known as the Dirac point (*V_Dirac_*), which is the switching point between the hole and electron carriers [29]. The pH sensitivity of the G-ISFET was evaluated by the shift of *V_Dirac_* in the pH buffer solution. The *V_Dirac_* was shifted by 19.4 mV/pH in the positive direction over the range of pH values from 4 to 10 in oxygenated G-ISFET, as shown in Figure 2c. There are some defects on the oxygenated graphene surface and edge, which are induced during oxygen plasma treatment. These defects, hydroxyl and carbonyl groups, can react with the protons in the electrolyte solution (protonation or deprotonation), leading to pH sensitivity in the oxygenated graphene. To confirm the reliability of the pH sensitivity in the oxygenated G-ISFET, *V_GS_* was swept with forward and backward bias to assess the *I_DS_–V_GS_* characteristics of the G-ISFET (Figure S2a,b). The *V_Dirac_* was shifted depending on the pH value in the buffer solution, regardless of the bias direction. However, hysteresis was shown along the *V_GS_* sweep direction (Figure S2c,d). This is because the mobility of the ions in the solution is slow, so the ions do not move quickly along the *V_GS_* sweep direction.

**Figure 3:**
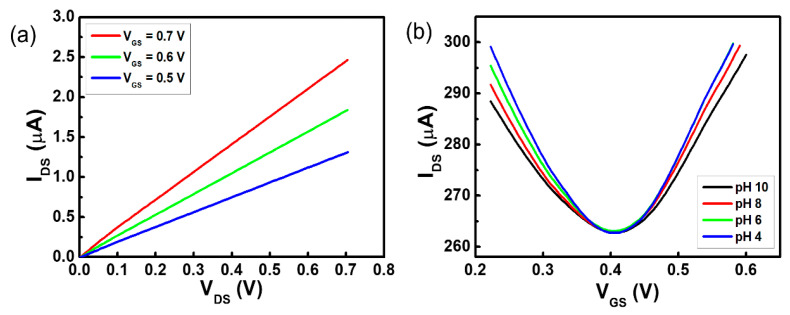
Characteristic graphs of fluorinated G-ISFET: (**a**) *I_DS_*–*V_DS_* transfer characteristic with respect to *V_GS_* and (**b**) *I_DS_*–*V_GS_* transfer characteristic with respect to pH value.

The conductivity of the fluorine-functionalized graphene sheet with a semi-ionic bond is that of a semiconductor [21]. To obtain the *I_DS_–V_DS_* characteristics of the fluorinated graphene channel in a buffer solution of pH 8, *V_DS_* was swept from 0.0 to 0.7 V. The fluorinated gate channel of the G-ISFET worked stably in the electrolyte solution and *I_DS_* increased depending on the value of *V_GS_* at the n-channel region, as shown in Figure 3a. However, *V_Dirac_* did not shift over the range of pH values from 4 to 10 in the fluorinated G-ISFET, as shown in Figure 3b. The fluorinated G-ISFET was insensitive to pH. The gate leakage current (*I_GS_*) of the fluorinated G-ISFET was 16.48 nA. The pH sensitivity was shown with a different F/C ratio on the fluorinated graphene surface (Figure S3). We typically conducted the *I_DS_–V_GS_* transfer characteristics of the fluorinated G-ISFET to assess the cation and anion sensitivity by KCl concentration and to evaluate the interfacial potential according to the ionic strength in Tris buffer solution. The fluorinated G-ISFET was insensitive to K^+^ and Cl^−^ ions and worked stably in Tris buffer solution regardless of ionic strength (Figure S4a,b). This fluorinated gate channel has the potential to be used as a reference electrode in pH-sensing devices to probe electrostatic potential in the electrolyte solution.

**Figure 4:**
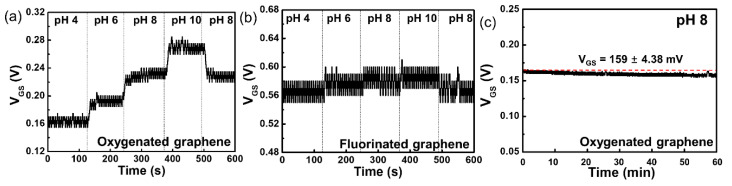
Real-time detection of pH in electrolyte solution using (**a**) oxygenated G-ISFET and (**b**) fluorinated G-ISFET. (**c**) Long-term stability of oxygenated G-ISFET in a buffer solution of pH 8.

The real-time pH sensitivity of the oxygenated G-ISFET with Ag/AgCl reference electrode was evaluated. *V_GS_* was continuously measured at fixed values of *I_DS_* and *V_DS_* on the *I_DS_–V_GS_* characteristics with periodic injection of buffer solutions with different pH values every 2 min for 10 min. *V_DS_* was maintained as constant, which was chosen so as to bias the device in strong inversion. The results of real-time measurements on the oxygenated G-ISFET with buffer solutions of different pH values are shown in Figure 4a. In the n-channel region, *V_GS_* increases in the high-pH buffer solution to maintain *I_DS_* (40 µA) at a fixed value of *V_DS_* (0.05 V) on the oxygenated gate channel because the surface charge on the oxygenated gate channel was negative, owing to deprotonation in the high-pH buffer solution. On the other hand, the surface charge is positive owing to protonation in the low-pH buffer solution, and *V_GS_* decreases to maintain *I_DS_* at the fixed value of *V_DS_*. The results of real-time measurements on the fluorinated G-ISFET in different pH buffer solutions are shown in Figure 4b. *V_GS_* was continuously measured at fixed values of *I_DS_* (150 µA) and *V_DS_* (0.05 V) in the *I_DS_–V_GS_* characteristics with periodic injection of buffer solutions with different pH values every 2 min for 10 min. The fluorinated G-ISFET was insensitive to pH, which was in agreement with the static characteristics.

We evaluated the long-term stability of oxygenated G-ISFET in a buffer solution of pH 8 in real-time, similar to the drift characteristics of the ISFET. *V_GS_* was continuously measured to keep *I_DS_* at 135 µA and *V_DS_* at 0.05 V in the *I_DS_–V_GS_* characteristics. *V_GS_* was continuously maintained at the voltage of 159 ± 4.38 mV for 60 min, as shown in Figure 4c.

### 3.3. Two-Channel G-SGFET

**Figure 5:**
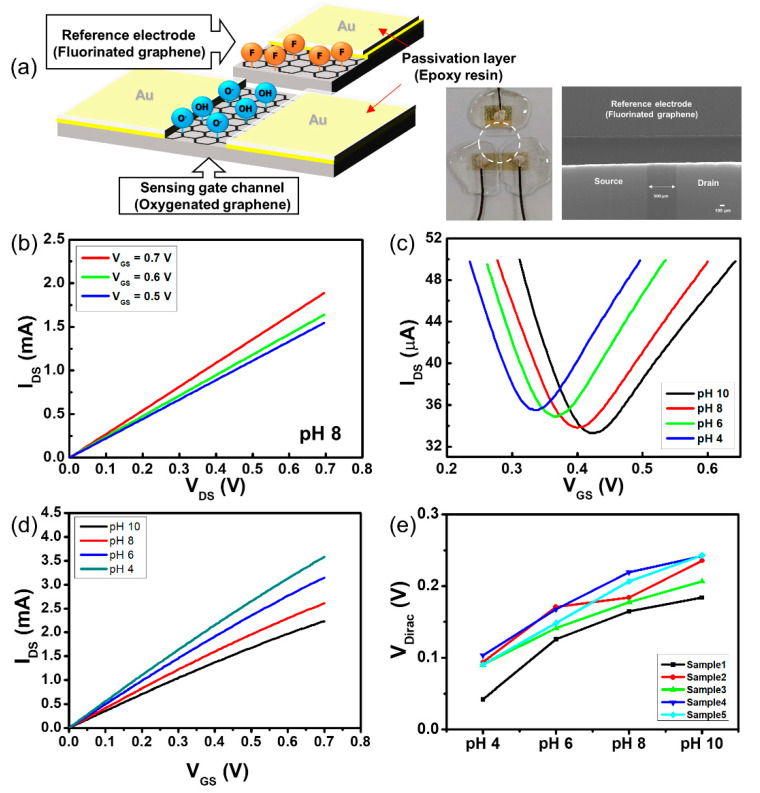
Two-dimensional pH sensor using graphene field-effect transistor. (**a**) Schematic illustration of two-channel graphene solution gate field-effect transistor (G-SGFET) and sensor image. For two-channel G-ISFET: (**b**) *I_DS_*–*V_DS_* transfer characteristic with respect to *V_GS_* and (**c**) *I_DS_*–*V_GS_* transfer characteristic with respect to pH value (**d**) *I_DS_–V_DS_* transfer characteristic with respect to pH value. (**e**) The Dirac point of the two-channel G-STGFET.

We fabricated a two-channel G-SGFET, as shown in Figure 5a. One channel is the oxygenated channel that serves as a sensing G-ISFET and the other channel is a fluorinated channel that serves as an Ag/AgCl reference electrode. The fluorinated graphene electrode was placed close enough to the sensing G-ISFET so that its fixed potential could control the operation of the G-ISFET. It should be noted that both the sensing channel and fluorinated graphene reference electrode were in direct contact with the electrolyte solution.

In the two-channel G-SGFET, *V_GS_* is the voltage between the fluorinated graphene reference electrode and the source electrode of G-ISFET (*V_FS_*), which is the same as when an Ag/AgCl reference electrode is used, as shown in Figure 3a. The *I_DS_–V_DS_* characteristics of the two-channel G-SGFET in a buffer solution of pH 8 are shown in Figure 5b. *V_DS_* was swept from 0.0 to 0.7 V and *I_DS_* was increased with respect to *V_GS_* (0.5, 0.6, and 0.7 V) in the n-channel region. To obtain the *I_DS_–V_GS_* characteristics of the two-channel G-SGFET in the pH buffer solution, *V_DS_* was fixed at 0.05 V and *V_GS_* was swept from 0.0 to 0.65 V. The transfer characteristics showed ambipolar graphene FET behavior (p-channel and n-channel), which is a typical characteristic curve of the G-ISFET, and *V_Dirac_* of the two-channel G-SGFET was shifted by 18.2 mV/pH in the positive direction over the range of pH values from 4 to 10, as shown in Figure 5c. The *I_DS_–V_DS_* characteristics of the two-channel G-SGFET in the pH buffer solution, *V_GS_* was fixed at 0.4 V and *V_DS_* was swept from 0.0 to 0.7 V, are shown in Figure 5d. *I_DS_* depended on the pH value in the electrolyte solution. We fabricated 5 samples to evaluate the reproducibility of pH sensitivity on the two-channel G-SGFET. The two-channel G-SGFET was sensitive to pH regardless of the sample, as shown in Figure 5e. The average pH sensitivity of the two-channel G-SGFET at pH 4–6 was 49.9 mV, at pH 6–8, the pH sensitivity was 33.5 mV, and at pH 8–10, the pH sensitivity was 28.8 mV. The pH sensitivity is high in the acidic region, which is similar to the use of Ag/AgCl reference electrode on the oxygenated G-ISFET. The Dirac point of the two-channel G-SGFET in the same pH solution varies from sample to sample because sensor samples are made manually at the lab level.

The voltage between the G-ISFET and the fluorinated graphene reference electrode was set with respect to the sensing channel and the reference electrode interface. Considering *V_FS_* in the fluorinated graphene reference electrode, the change in the surface charge in the sensing channel results in the variation of the voltage between the sensing channel and the fluorinated graphene reference electrode. The bulk potential of the solution is determined by *V_FS_* in the fluorinated graphene reference electrode with electrostatic equilibrium and capacitive coupling. Therefore, the voltage between the sensing channel and the fluorinated graphene reference electrode is the only parameter related to the concentration of protons ([H^+^]) in the electrolyte solution. The change in proton concentration in the electrolyte solution leads to the variation of the surface charge by protonation or deprotonation on the sensing channel and modulates the channel conductance of the oxygenated channel in the two-channel G-SGFET. The variation of *V_Dirac_* on the two-channel G-SGFET can be expressed as follows:

Δ*V*_*Dirac*_ = (*V*_*pHO*_ − *V*_*S*_) − (*V*_*pHF*_ + *V*_*F*_ − *V*_*S*_) (1)

where *V_pHO_* is the pH sensitivity of the oxygenated sensing channel, *V_S_* is the potential of the source electrode, *V_pHF_* is the pH sensitivity of the fluorinated graphene reference electrode, and *V_F_* is the potential of the fluorinated graphene reference electrode in the two-channel G-SGFET. The pH sensitivity of the two-channel G-SGFET is determined by the differential response between the oxygenated sensing channel (*V_pHO_*) and the fluorinated reference electrode (*V_pHF_*). The pH sensitivity of the two-channel G-SGFET is lower than when the Ag/AgCl reference electrode is used because the fluorinated graphene reference electrode has some defect, such as an sp^3^-hybridized carbon structure (3.4% covalent bond) and an amorphous carbon structure. These defects are unstable in the electrolyte solution.

**Figure 6:**
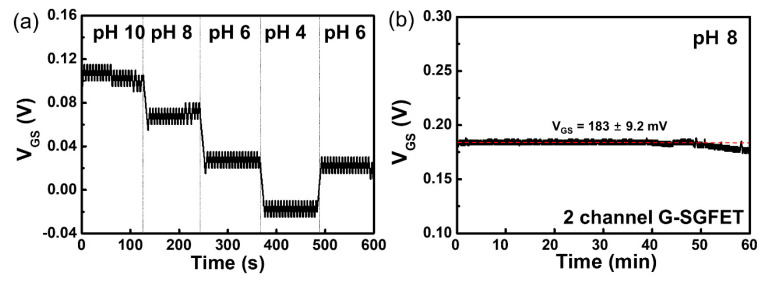
(**a**) Real-time detection of pH in electrolyte solution using two-channel G-SGFET. (**b**) The long-term stability of two-channel G-SGFET in a buffer solution of pH 8.

The real-time pH sensitivity of the two-channel G-SGFET was measured over the range of pH values from 4 to 10. *V_GS_* was continuously measured at the fixed values of *I_DS_* (150 µA) and *V_DS_* (0.05 V) on the *I_DS_–V_GS_* characteristic with periodic injection of buffer solutions with different pH values every 2 min for 10 min. These conditions were the same as that when an Ag/AgCl reference electrode was used. The real-time pH sensitivity and hysteresis of the two-channel G-SGFET with different pH buffer solutions was shown in Figure 6a and Figure S5a. In the n-channel region, *V_GS_* increased in the high-pH buffer solution to maintain *I_DS_* at a fixed value of *V_DS_* in the two-channel G-SGFET because the surface charge of the oxygenated gate channel became negative by deprotonation in the high-pH buffer solution. On the other hand, the surface charge is positive by protonation in the low-pH buffer solution and *V_GS_* decreases to maintain *I_DS_* at a fixed value of *V_DS_*. We evaluate the long-term stability of the two-channel G-SGFET in the buffer solution of pH 8 in real-time. *V_GS_* was continuously measured to keep *I_DS_* (130 µA) at a fixed value of *V_DS_* (0.05 V) in the *I_DS_–V_GS_* characteristics. *V_GS_* is continuously maintained at the voltage of 183 ± 9.2 mV for 60 min, which is similar to using an Ag/AgCl electrode, as shown in Figure 6b. However, the stability of two-channel G-SGFET was decreased after 1 h, as shown in Figure S5b. The stability of the two-channel G-SGFET is lowered after 1 h, but within 1 h, the pH sensitivity is stable. Therefore, it is expected to be fully utilized as a disposable pH sensor.

To achieve high pH sensitivity, it is critical for the sensing channel of the two-channel G-SGFET to have an ideal *Nernstian* response while the reference electrode remains entirely insensitive to pH. We adopted the partially oxygenated graphene electrode as a sensing channel with pH sensitivity of 19.4 mV/pH, whereas the semi-ionic C-F bonding graphene electrode was chosen as a reference electrode in the two-channel G-SGFET. When the plasma treatment time increased in the oxygen gas environment, the pH sensitivity of the two-channel G-SGFET increased as the number of binding sites of [H^+^] increased on the oxygenated gate channel. However, the large degree of surface modification using plasma treatment, the resistance of the oxygenated gate channel increased and the two-channel G-SGFET unstably worked in electrolyte solution, because the surface of the graphene layer was etched by oxygen plasma [29].

## 4. Conclusions

Oxygenated and fluorinated graphene were directly exposed to the electrolyte solution for sensing pH and serving as the reference electrode in the two-channel G-SGFET, respectively. The transfer and output characteristics of the two-channel G-SGFET in the electrolyte solution were the same as those obtained when an Ag/AgCl reference electrode was used on the G-ISFET. The pH sensitivity in the two-channel G-SGFET was determined by the differential response between the oxygenated graphene sensing channel and the fluorinated reference electrode, which worked stably in the electrolyte solution. The pH sensor of the two-channel G-SGFET has the potential to be applied as an implantable pH sensor by utilizing the biocompatibility of graphene.

To work as a sensor in the electrolyte solution, the ISFET needs a stable reference electrode. Generally, an Ag/AgCl electrode is used as a reference electrode in the sensing system. In this work, we have proposed a new reference electrode that is compatible with semiconductor fabrication technology because the reference electrode was fabricated using the same material as the sensing channel using graphene electrode.

If the fluorinated graphene electrode can be used as the reference electrode by replacing the Ag/AgCl reference electrode in the existing pH sensor system, the application range of the pH sensor can be further expanded through the manufacture of a compact and disposable pH sensor.

## Supplementary Materials

The following are available online at https://www.mdpi.com/1424-8220/20/15/4184/s1, Figure S1. (a) Deconvoluted C 1s peaks on pristine graphene and (b) partially fluorinated graphene. Figure S2. *I_DS_-V_GS_* transfer characteristic of G-ISFET according to forward and backward gate bias (−0.1 to 0.4). (a) *I_DS_-V_GS_* transfer characteristic with pH value with the forward and (b) backward gate bias. (c) *I_DS_-V_GS_* transfer characteristic of G-ISFET with forward and backward bias at pH 6 and (d) pH 8. Figure S3. XPS spectra and *I_DS_-V_GS_* transfer characteristic of fluorinated graphene due to increase plasma time. As the plasma time increased (5 min, 20 min), the fluorine atomic ratio of fluorinated graphene was increased ((a) 28.7% and (c) 49.85%). However, the pH sensitivity of fluorinated graphene was decreased ((b) 10.5 and (d) 1.28 mV/pH) according to the plasma time. Figure S4. *I_DS_-V_GS_* transfer characteristic of fluorinated graphene (FG) due to the anion concentration and ionic strength. (a) *I_DS_-V_GS_* transfer characteristic of fluorinated graphene due to different concentration of KCl solution. (b) *I_DS_-V_GS_* transfer characteristic of fluorinated graphene due to different ionic concentration of Tris-HCl solution. Figure S5. Real-time detection of pH in electrolyte solution using the two-channel G-SGFET. (a) The hysteresis characteristics of the two-channel G-SGFET from pH 4–10–4. (b) The stability of the two-channel G-SGFET in a buffer solution of pH 8 for 6 h (*V_DS_* = 0.05V, *I_DS_* = 130 µA). Video S1: The video file is the actual experimental environment.
