# Supplementary material for: Two-Channel Graphene pH Sensor Using Semi-Ionic Fluorinated Graphene Reference Electrode
Source: Sensors (Basel). 2020 Jul 28;20(15):4184. doi: 10.3390/s20154184 (PMC7436108; doi:10.3390/s20154184)
Supplement: Supplementary file 1 [file sensors-20-04184-s001.pdf]

# Supplementary Information

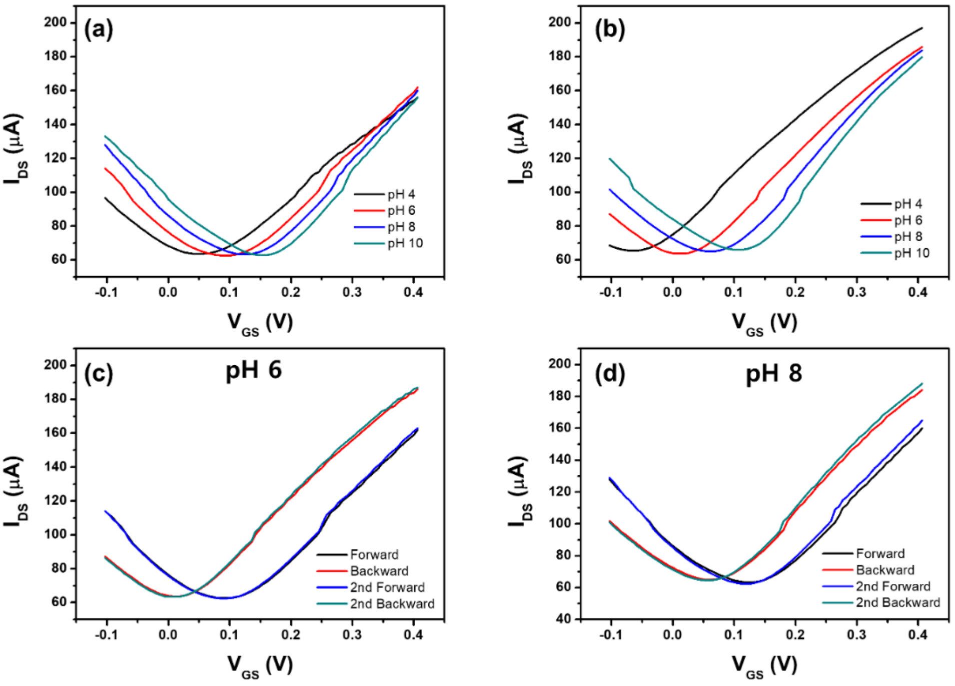


Figure 1. The $I_{DS}$-$V_{GS}$ transfer characteristic of G-ISFET according to forward and backward gate bias (-0.1 to 0.4). (a) The $I_{DS}$-$V_{GS}$ transfer characteristic with pH value with the forward and (b) backward gate bias. (c) The $I_{DS}$-$V_{GS}$ transfer characteristic of G-ISFET with forward and backward bias at pH 6 and (d) pH 8.

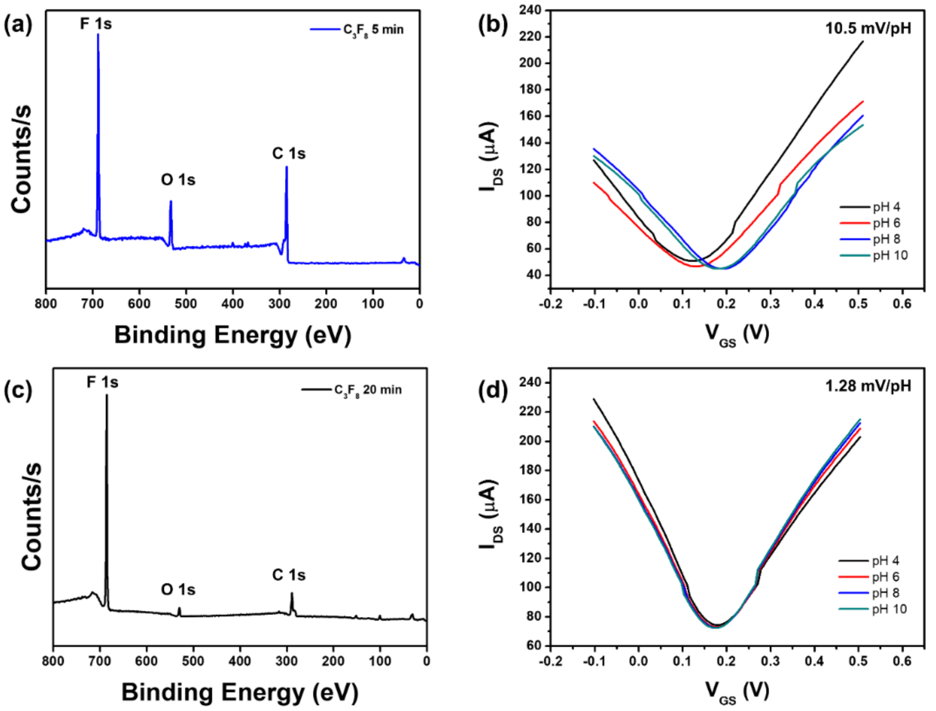


Figure S2 The XPS spectra and $I_{DS}$-$V_{GS}$ transfer characteristic of fluorinated graphene due to increase plasma time. As the plasma time increased (5 min, 20 min), the fluorine atomic ratio of fluorinated graphene was increased ((a) 28.7% and (c) 49.85%). However, the pH sensitivity of fluorinated graphene was decreased ((b) 10.5 and (d) 1.28 mV/pH) according to the plasma time.

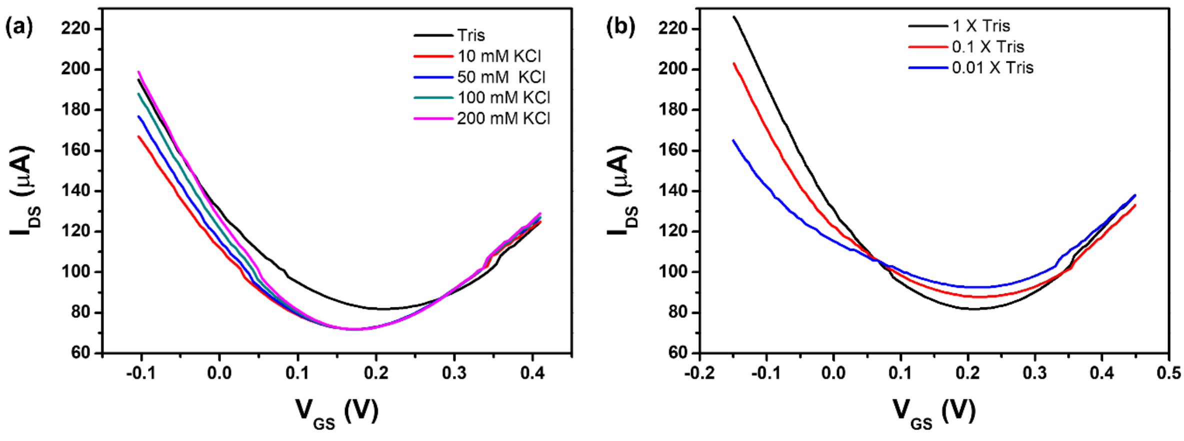


Figure S3. The $I_{DS}$-$V_{GS}$ transfer characteristic of fluorinated graphene (FG) due to the anion concentration and ionic strength. (a) The $I_{DS}$-$V_{GS}$ transfer characteristic of fluorinated graphene due to different concentration of KCl solution. (b) The The $I_{DS}$-$V_{GS}$ transfer characteristic of fluorinated graphene due to different ionic concentration of Tris-HCl solution.

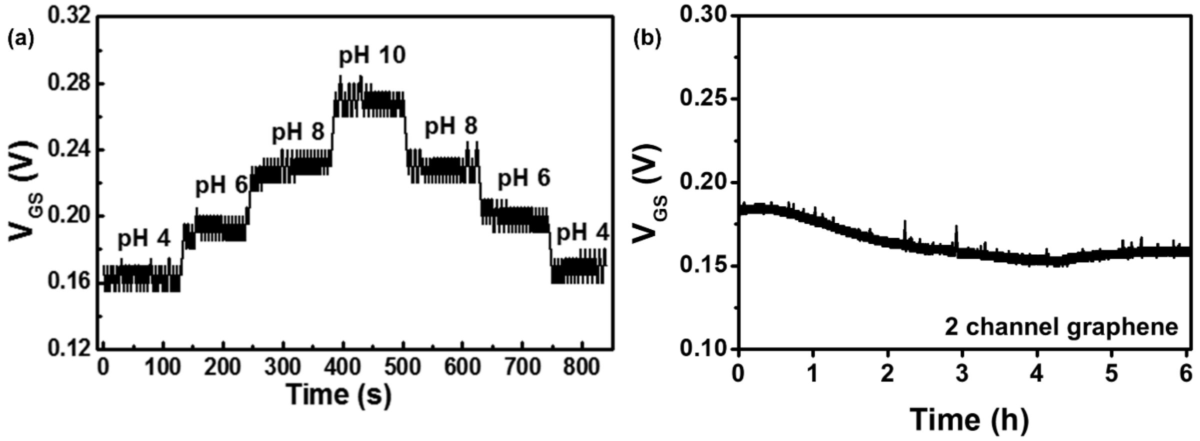


Figure S4. Real-time detection of pH in electrolyte solution using the two-channel G-SGFET. (a) The hysteresis characteristics of the two-channel G-SGFET from pH 4–10–4. (b) The stability of the two-channel G-SGFET in a buffer solution of pH 8 for 6 h ($V_{DS}$ = 0.05V, $I_{DS}$ = 130 μA).
